# Studying different variations of ranking method to explore health related quality of life dimensions prioritization

**DOI:** 10.1007/s11136-026-04350-5

**Published:** 2026-08-01

**Authors:** Yanna Zhou, Yifan Ding, Jan Busschbach, Xiuquan Shi, Zhihao Yang

**Affiliations:** 1 Department of Epidemiology and Health Statistics, School of Public Health, Zunyi Medical University, Zunyi, Guizhou China; 2 Section Medical Psychology and Psychotherapy, Department of Psychiatry, Erasmus MC, University Medical Center Rotterdam, Rotterdam, The Netherlands; 3 Health Services Management Department, Guizhou Medical University, Guiyang, China

**Keywords:** Health-related quality of life, EQ-5D, Culture, Preference, Ranking method

## Abstract

**Purpose:**

Researchers have argued that EQ-5D may not capture all aspects in health-related quality of life (HRQoL), they therefore proposed adding additional dimensions. However, whether additional dimensions are more important than EQ-5D dimensions is unknown. This study aimed to explore the impact of four different ranking tasks on eliciting health preferences and assess which ranking task provides better consistency and test–retest reliability.

**Methods:**

Besides the five EQ-5D dimensions, nine HRQoL dimensions were selected, including culturally universal dimensions and culturally specific dimensions relevant to Chinese. To evaluate the impact of dimension descriptions and instructions, we tested four ranking tasks derived from two dimension description versions and two instruction versions. Each participant completed the questionnaires twice, enabling intra-group comparisons, including test–retest reliability when using same questionnaire and consistency when using different questionnaires.

**Results:**

The phrase “moderate problems with [dimension]” yielded more stable rankings than simply using “problems”, even when the instruction varied. When dimension descriptions differed, the correlation between rankings was lower (*rₛ* = 0.859) than when instructions varied (*rₛ* = 0.961). Additionally, when using the same instruction, aggregate test–retest reliability of “moderate problems” was higher (*rₛ* = 0.972) than that of “problems” (*rₛ* = 0.871). The average median of correlation coefficient of “moderate problems” (0.81 (0.56~0.89)) was higher than that of “problems” (0.70 (0.45~0.79)).

**Conclusion:**

This study shows that ranking method is feasible and that the dimension description influences the outcome. When compared to “problems” description, the results using “moderate problems” description shows higher aggregate stability and individual-level test–retest consistency.

**Supplementary Information:**

The online version contains supplementary material available at https://doi.org/10.1007/s11136-026-04350-5.

## Introduction

The EQ-5D was developed by the EuroQol Group to provide a concise, generic instrument and to ensure comparability between the results of different studies. The researchers made the first choice of the dimensions based on a review of other available generic health instruments [1]. At first, there were six dimensions: Mobility, daily activities and self-care, psychological functioning, social and role performance, and pain or other health problems [2]. Based on further empirical tests, the instrument was refined to five dimensions: Mobility, self-care, usual activities, pain/discomfort and anxiety/depression.

Though EQ-5D is one of the most widely applied health status instrument [1], scholars argued that limited number of dimensions may not capture all aspects of health-related quality of life (HRQoL). Therefore, some researchers have proposed adding bolt-on dimensions based on clinical needs or cultural considerations[3], such as skin irritation for skin disease [4, 5], sleep and appetite for Chinese [6, 7], and “interpersonal relationships” and “activities related to bending knees” for Thai people [8, 9].

Increasing the number of dimensions will most likely improve sensitivity and enhance relevance for specific cultures [10, 11]. However, increasing the number of dimensions may reduce comparability with existing results, oblige the development of new value sets, increase the complexity and cost of these valuation studies [12]. It also remains an open question whether culture is indeed a major determinant in the selection of HRQoL dimensions. For instance, the content of EQ-5D [13] and EQ-HWB (experimental version) [14] could reflect the quality of life perceptions of Chinese individuals.

All the above suggests that adding more dimensions to improve sensitivity is insufficient to justify the associated costs. A more compelling justification would require evidence that the proposed additional dimensions are considered more relevant to HRQoL than EQ-5D dimensions. This could be tested by asking respondents to rank both EQ-5D dimensions and the proposed additional dimensions. If the additional dimensions clearly rank above the EQ-5D dimensions, this will suggest that they are more relevant to HRQoL.

The ranking of the HRQoL dimensions can be conducted in numerous ways. A full-scale valuation using Time Trade-Off or Discrete Choice Experiments is considered the most comprehensive. However, such methods are expensive and complex, and differences in outcomes across studies are largely driven by the operation of local research teams [15]. Therefore, a straightforward, standardized and reliable approach is needed to compare the importance of HRQoL dimensions and enable reproducible cross-cultural comparisons. Although ranking method is limited in its ability to reflect utility magnitude, it offers a low-burden approach for analyzing preferences of health dimensions. Importantly, the research question is whether this group of dimensions is perceived as most prominent than the EQ-5D core dimensions. Therefore, we hope to develop a ranking task to instruct participants to rank health dimensions, rather than to rank complete health states or combinations of dimension levels. However, there is currently no standardized ranking task, neither in terms of instructions nor in terms of dimension descriptions.

To address this gap and facilitate future cross-cultural comparisons, this study was conducted to examine the influence of two variations within the ranking task: (1) variation in the instructions, and (2) variation in dimension descriptions. The following questions were tested: (1) Whether variations in ranking tasks influence the outcomes; (2) Which ranking task yields more valid results. Consistency across different ranking tasks and test–retest reliability of the same ranking task were used as evaluation metrics.

## Methods

### Ethical approval

The study received ethical approval from the Health Services Management Department, Guizhou Medical University (2024–40).

### Selecting dimensions

Besides the EQ-5D dimensions, we selected nine additional dimensions based on a narrative review followed by group discussion. Mao [6] and Fan [7] proposed seven bolt-on dimensions that would be relevant for measuring HRQoL in Chinese cultural context, including sleep, tiredness/lack of strength, appetite, emotional control, social adaptation, social support and climate adaptation. Ding [14] reported that vision and cognition were also highly rated among Chinese respondents. These two dimensions are frequently mentioned as bolt-ons for the EQ-5D in broader literature [3, 16]. The translations of these dimensions were validated by the bilingual team members (YN, YF and ZH).

### Classifying dimensions

Among the nine selected dimensions, emotional control, social adaptation and climate adaptation were mentioned exclusively in relation to Chinese culture [7, 17], and were therefore classified as “culturally specific dimensions”. Appetite, sleep, cognition, vision, social support and tiredness/lack of strength were mentioned not only in Chinese literature but also in literature based on Western populations [18]. Notably, these six dimensions were also selected as bolt-ons in Western general population samples [11], supporting their classification as “culturally universal dimensions”. Appetite, sleep, cognition, vision, and tiredness/lack of strength reflect physical or sensory functions and are related to physiological needs [19]. When physiological needs are realized, the need for safety and security will emerge. Therefore, we classified these dimensions as physical and sensory functions and hypothesize that people may rank these basic functions highly. See in Table 1.

**Table 1:** Dimensions of health-related quality of life (HRQoL)

Category	Dimensions
Original EQ-5D dimensions	1. Mobility (walking about)
	2. Self-Care (such as washing or dressing by myself)
	3. Usual activities (such as work, study, housework, family or leisure activities)
	4. Pain/discomfort
	5. Depression/anxiety
Additional culturally specific dimensions: Truly local concepts of HRQoL, specific to a culture or region, (e.g., emotional control” or climate adaptation in a Chinese context)	6. Adaptation to society
	7. Emotional control
	8. Climate adaptation
Additional culturally universal dimensions: relevant across many cultures but may not be explicitly included in the EQ-5D instrument, (e.g., social relationships)	9. Social support (relationships)
	10. Cognition (memory decline or inability to concentrate)
	11. Appetite
	12. Tiredness/lack of strength
	13. Vision (seeing)
	14. Sleep (difficulty initiating / maintaining sleep / undesired early awakenings with associated daytime impairment)

The 10th to 14th dimensions are basic physical or sensory functions

### Ranking method

A pilot study was conducted to evaluate the feasibility of ranking method and provide an initial rankings (more details is provided in the Online Resource 1). Then we randomized the pilot study’s initial rankings to generate a fixed sequence. This fixed sequence was used in subsequent studies, with only the problem levels of the dimension description being modified. Participants were presented with a list of 14 dimensions (an example is provided in the Online Resource 2 Figure S1) and were instructed to rank them from “first to cure” to “last to cure”. They could drag the dimensions, placing the highest priority at the top and the lowest at the bottom. Participants could adjust the order before submitting their final response. The final order was recorded for analysis.

### Variations in dimension descriptions

Given that the aim was to explore preferences for health dimensions rather than severity levels within each dimension, a consistent severity level was applied across all dimensions. Two types of dimension-level descriptions were developed. The first used the phrase “moderate problems” for each dimension (e.g., “moderate problems with sleep”), according to the third level of the EQ-5D. The second used a simpler phrase (e.g., “problems with sleep”). All descriptions were framed negatively to maintain consistency.

### Variations in instructions

In addition to the variation in dimension descriptions, two instruction versions were tested. These were inspired by two established preference elicitation methods: ranking (ranking all alternatives simultaneously) [20] and the Kaizen-task (repeatedly selecting one alternative to improve) [21].

The first version (Short Version) was based on ranking method, asking participants to rank all dimensions from highest to lowest priority for cure. The second version (Long Version) was inspired by the Kaizen-task. In Craig’s research [21], researchers guided participants to repeatedly select one dimension they would prefer to improve first from the remaining options, continuing this process until a complete preference order was established. In our study, we used the word “cure” instead of “improve” because the task concerned the complete removal of a health problem rather than partial improvement. Additionally, the health problem that one wishes to cure first is the one perceived to have the greatest impact on HRQoL, reflecting the real-world impact of health conditions on patients’ lives [22]. The instructions were provided in Online Resource 3.

Four different questionnaires were developed (See Online Resource 3) and four groups were recruited accordingly. The first administration is presented in Table 2.

**Table 2:** Study design: Group allocation and sequence of administration

Group	First administration	Second administration	Subjects comparison
1	Q1: Problems + Long Version	Q3: Moderate problems + Long Version	Problems versus Moderate problems in Long Version
2	Q3: Moderate problems + Long Version	Q4: Moderate problems + Short Version	Long Version versus Short Version in moderate problems
3	Q2: Problems + Short Version	Q2: Problems + Short Version	Test–retest
4	Q4: Moderate problems + Short Version	Q4: Moderate problems + Short Version	Test–retest

Q means questionnaire

### Exploring correlations and test–retest reliability

After one week, participants were invited to complete the questionnaires again. We hypothesized that the differences between instructions would be less influential than the differences in dimension descriptions. Therefore, test–retest reliability for long version instruction was not assessed. Instead, the retest was conducted to assess the impact of variations in both dimension descriptions and instructions (See Table 2, groups 1 and 2). Additionally, by using each of the two predefined combination**—**short version with the phrasing “problems” and short version with “moderate problems”**—**twice, test–retest reliability could be assessed for both dimension-level descriptions (See Table 2, groups 3 and 4).

### Participants and Data Collection

Informed consent was obtained from all participants in accordance with ethical requirements. In June 2025, we recruited 8 students as our interviewers, 4 males and 4 females. Each interviewer completed one ranking task. They were encouraged to invite their friends to participate and complete the ranking task, following a snowball sampling approach, resulting in a convenience sample. Furthermore, because recruitment was conducted through interviewer-led snowball sampling within naturally occurring student networks, random assignment of participants to different administrations was not feasible in practice and would have considerably complicated the data collection process. Thus, group assignment was nonrandom. The target sample size was informed by previous research [20], with the aim of recruiting 10 women and 10 men for each group. Finally, 81 undergraduates from Zunyi Medical University were recruited. We assumed that they had no prior experience in quality-of-life research.

Given that undergraduates are generally well-educated and able to complete the questionnaire independently, data were collected through a self-reported method. The interviewer reminded the participants only need to trade off among dimensions, without consideration of treatment or intervention. Besides demographic characteristics (sex and age), the EQ-5D-5L and the EQ VAS (EuroQol Visual Analogue Scale) were administrated to illustrate the health of the participants. The Wenjuanxing platform was used to collect data: https://www.wjx.cn/.

### Data analysis

SPSS 29.0 (IBM Corp., Armonk, NY, USA) was used for statistical analyses. Proportions were used to describe count data, and the Chi-square test was used to examine the differences between groups. Means and standard deviations **(**$\overline{{\boldsymbol{x}}}\pm {\boldsymbol{s} }$**)** were used to describe age and EQ VAS scores, and one way ANOVA was used to evaluate the differences between groups. The mean rank, median rank, and 95% Bootstrap Confidence Interval (CI) with 1000 resamples were used to evaluate the importance of each dimension. A “high” ranking indicates that an item is selected first, which appears as a low-ranking score. Spearman correlation coefficient was used to evaluate the consistency of rankings and test–retest reliability. The test–retest reliability includes group level reliability and individual level reliability. Since the spearman correlation coefficients were non-normal distribution, Median and Interquartile Range (M, IQR) were used to describe coefficients of the individual level reliability. The statistically significant level was 0.05.

## Results

### Demographic characteristics and health status

Groups 1, 2 and 3 each included 20 students, while group 4 included 21 students. The average age **(**$\overline{{\boldsymbol{x}}}\pm {\boldsymbol{s} }$**)** for groups 1 to 4 were (19.75 ± 0.79), (21.10 ± 0.55), (19.15 ± 0.93) and (21.19 ± 0.81) years, respectively, with significant differences between groups (*F* = 33.587, *P* < 0.001). The proportions of men in groups 1 to 4 were 50.0%, 50.0%, 55.0% and 52.4%, respectively, without significant differences between groups (${\chi }^{2}$= 0.137, *P* = 0.987). As expected in a young student population, only a few students reported slight problems or moderate problems in mobility or usual activity, while the most reported issues were anxiety/depression and pain/discomfort. The average EQVAS scores **(**$\overline{{\boldsymbol{x}}}\pm {\boldsymbol{s} }$**)** for groups 1 to 4 were (84.05 *± * 9.78), (82.65 *± * 10.66), (75.35 *± * 12.26) and (79.71 *± * 19.87), respectively, without significant differences between groups (*F* = 1.546, *P* = 0.209). (See Online Resource 4 Table S1).

### The impact of different dimension descriptions and instructions as measured between groups in first administration

Almost 50% of respondents, and even more, put the top 5 ranked dimensions in the top 5 (See the Online Resource 4 Table S2 and Table S3). Table 3 presents the rankings from the first administration for four groups. In these between-subject results, columns 1 and 2 indicated that the type of instruction influenced the rankings when dimensions were phrased as “problems with [dimension]. For instance, vision was ranked first in column 1, while it dropped to 10 in column 2. Additionally, the spearman correlation coefficient between columns 1 and 2 was 0.525 (*P* > 0.05). However, when the dimensions were described as “moderate problems with [dimension]”, the difference in instructions had little influence on the rankings (see columns 3 and 4). The spearman correlation coefficient between columns 3 and 4 was 0.934 (*P* < 0.05). Thus, using the “moderate problems” phrasing appeared to produce more stable results, even different instructions were used.

**Table 3:** The rank order changes of 4 types of questionnaires for first administration in different groups

	Group 1 and 3: Difference in instruction description: Long Version versus Short Version. Both use the problems with [dimension] ( ${r}_{s}=$ 0.525)				Group 2 and 4: Difference in instruction description: Long Version versus Short version. Both use the moderate problems with [dimension] ( ${r}_{s}=$ 0.934^**^)			
Number	Group 1 (n = 20) Questionnaire 1: Problems + Long Version	Mean rank, Median rank, 95% Bootstrap CI	Group 3 (n = 20) Questionnaire 2: Problems + Short Version	Mean rank, Median rank, 95% Bootstrap CI	Group 2 (n = 20) Questionnaire 3: Moderate problems + Long Version	Mean rank, Median rank, 95% Bootstrap CI	Group 4 (n = 21) Questionnaire 4: Moderate problems + Short Version	Mean rank, Median rank, 95% Bootstrap CI
1	Vision	3.7, 3.0 (2.6 ~ 4.9)	***Self-care***	4.4, 4.0 (3.1 ~ 6.0)	***Mobility***	5.2, 4.5 (3.8 ~ 6.8)	***Mobility***	3.7, 3.0 (2.6 ~ 5.0)
2	***Mobility***	5.1, 3.5 (3.4 ~ 6.9)	Sleep	5.8, 6.0 (4.5 ~ 7.1)	***Pain/discomfort***	5.2, 4.5 (3.7 ~ 6.8)	***Pain/discomfort***	4.8, 4.0 (3.2 ~ 6.4)
3	**Emotional control**	5.8, 5.5 (4.3 ~ 7.4)	***Mobility***	6.2, 6.5 (4.2 ~ 8.2)	***Self-care***	5.2, 5.0 (3.9 ~ 6.6)	***Self-care***	5.1, 4.0 (3.6 ~ 6.9)
4	Cognition	6.6, 6.5 (5.2 ~ 8.2)	***Usual activities***	6.3, 5.5 (4.6 ~ 8.0)	Sleep	5.4, 4.5 (4.1 ~ 6.8)	Cognition	6.0, 5.0 (4.4 ~ 7.8)
5	***Self-care***	7.0, 6.5 (5.2 ~ 8.8)	***Pain/discomfort***	6.4, 5.0 (4.8 ~ 8.2)	***Usual activities***	5.7, 6.0 (4.2 ~ 7.1)	Sleep	6.7, 7.0 (5.5 ~ 7.9)
6	***Depression/anxiety***	7.4, 8.0 (5.7 ~ 9.0)	Cognition	6.9, 7.5 (5.4 ~ 8.3)	Cognition	5.8, 6.0 (4.8 ~ 6.9)	***Depression/anxiety***	7.1, 7.0 (5.7 ~ 8.6)
7	***Pain/discomfort***	7.5, 7.5 (5.9 ~ 9.2)	**Emotional control**	6.9, 7.0 (5.4 ~ 8.4)	***Depression/anxiety***	6.7, 6.5 (4.8 ~ 8.7)	**Emotional control**	7.5, 8.0 (6.5 ~ 8.6)
8	Sleep	7.6, 6.5 (6.2 ~ 9.0)	***Depression/anxiety***	7.6, 7.5 (6.4 ~ 8.9)	Vision	6.9, 6.0 (4.8 ~ 8.9)	***Usual activities***	7.5, 7.0 (5.7 ~ 9.2)
9	Tiredness/lack of strength	8.1, 9.0 (6.5 ~ 9.6)	Social support (relationships)	7.8, 7.5 (6.2 ~ 9.5)	**Emotional control**	8.3, 9.5 (6.7 ~ 9.7)	Vision	7.6, 7.0 (5.7 ~ 9.6)
10	***Usual activities***	8.2, 9.0 (6.3 ~ 9.9)	Vision	8.1, 10.5 (5.8 ~ 10.3)	Appetite	9.0, 9.5 (7.3 ~ 10.6)	**Adaptation to society**	8.7, 9.0 (7.3 ~ 10.0)
11	**Adaptation to society**	8.3, 8.0 (7.1 ~ 9.5)	**Adaptation to society**	8.8, 9.5 (7.4 ~ 10.2)	**Adaptation to society**	9.3, 10.0 (7.9 ~ 10.6)	Social support (relationships)	8.9, 9.0 (7.5 ~ 10.2)
12	Social support (relationships)	8.7, 9.0 (7.3 ~ 10.0)	Tiredness/lack of strength	9.2, 9.5 (7.6 ~ 10.6)	Tiredness/lack of strength	9.9, 11.0 (8.8 ~ 10.9)	Tiredness/lack of strength	9.4, 10.0 (7.8 ~ 10.8)
13	Appetite	9.2, 9.5 (7.7 ~ 10.7)	Appetite	10.0, 10.0 (8.4 ~ 11.3)	Social support (relationships)	10.3, 11.0 (8.9 ~ 11.5)	Appetite	10.6, 11.0 (9.1 ~ 11.8)
14	**Climate adaptation**	12.2, 14.0 (10.7 ~ 13.4)	**Climate adaptation**	11.0, 11.5 (9.5 ~ 12.3)	**Climate adaptation**	12.7, 14.0 (11.7 ~ 13.6)	**Climate adaptation**	11.5, 13.0 (10.0 ~ 12.9)

***Bold italics*** means dimensions from EQ-5D, **bold** means culturally specific dimensions. The ranking order is from 1 (the most important) to 14 (the least important). **means *P* < 0.01

### The impact of different dimension descriptions and instructions between groups for two administrations

Table 4 presents the outcomes of group 1 and group 2 for the two administrations. Group 1 (columns 1 and 2) varied in different dimension description: Problems versus moderate problems, both employing long version instruction. The Spearman correlation coefficient of the mean rank in columns 1 and 2 was 0.859 (*P* < 0.01). In addition, the spearman correlation coefficients of questionnaire 1 (problems + long version) in group 1 and questionnaire 3 (moderate problems + long version) in group 2 were 0.594 (*P* < 0.05) (See Online Resource 4 Table S4).

**Table 4:** The rank order changes of different dimension descriptions and instructions in same groups for two administrations

Number	Group 1 (n = 20): Difference in level description: Problems versus moderate problems with [dimension]. Both use the Long Version ( ${r}_{s}=$ 0.859^**^)				Group 2 (n = 20): Difference in instruction: Long Version versus Short Version. Both use moderate problems with [dimension] ( ${r}_{s}=$ 0.961^**^)			
	First administration		Second administration		First administration		Second administration	
	Questionnaire 1: Problems + Long Version	Mean rank, Median rank, 95% Bootstrap CI	Questionnaire 3: Moderate problems + Long Version	Mean rank, Median rank, 95% Bootstrap CI	Questionnaire 3: Moderate problems + Long Version	Mean rank, Median rank, 95% Bootstrap CI	Questionnaire 4: Moderate problems + Short Version	Mean rank, Median rank, 95% Bootstrap CI
1	Vision	3.7, 3.0 (2.6 ~ 4.9)	***Mobility***	5.0, 3.5 (3.3 ~ 7.0)	***Mobility***	5.2, 4.5 (3.8 ~ 6.8)	***Mobility***	4.6, 3.5 (3.1 ~ 6.5)
2	***Mobility***	5.1, 3.5 (3.4 ~ 6.9)	Vision	5.4, 3.5 (3.7 ~ 7.4)	***Pain/discomfort***	5.2, 4.5 (3.7 ~ 6.8)	***Self-care***	4.8, 3.5 (3.3 ~ 6.7)
3	**Emotional control**	5.8, 5.5 (4.3 ~ 7.4)	***Depression/anxiety***	6.0, 7.0 (4.7 ~ 7.3)	***Self-care***	5.2, 5.0 (3.9 ~ 6.6)	***Pain/discomfort***	5.3, 4.0 (3.8 ~ 7.1)
4	Cognition	6.6, 6.5 (5.2 ~ 8.2)	***Self-care***	6.1, 5.0 (4.3 ~ 8.2)	Sleep	5.4, 4.5 (4.1 ~ 6.8)	***Usual activities***	5.4, 4.5 (3.8 ~ 7.1)
5	***Self-care***	7.0, 6.5 (5.2 ~ 8.8)	***Pain/discomfort***	6.3, 6.0 (4.7 ~ 7.9)	***Usual activities***	5.7, 6.0 (4.2 ~ 7.1)	Sleep	5.7, 5.0 (4.5 ~ 7.1)
6	***Depression/anxiety***	7.4, 8.0 (5.7 ~ 9.0)	Sleep	6.6, 6.0 (5.1 ~ 8.1)	Cognition	5.8, 6.0 (4.8 ~ 6.9)	Cognition	6.8, 7.0 (5.7 ~ 7.8)
7	***Pain/discomfort***	7.5, 7.5 (5.9 ~ 9.2)	**Emotional control**	7.6, 7.5 (5.8 ~ 9.2)	***Depression/anxiety***	6.7, 6.5 (4.8 ~ 8.7)	***Depression/anxiety***	7.0, 7.0 (5.6 ~ 8.3)
8	Sleep	7.6, 6.5 (6.2 ~ 9.0)	Cognition	7.8, 8.0 (6.3 ~ 9.3)	Vision	6.9, 6.0 (4.8 ~ 8.9)	Vision	7.9, 6.0 (6.1 ~ 10.0)
9	Tiredness/lack of strength	8.1, 9.0 (6.5 ~ 9.6)	***Usual activities***	7.9, 9.0 (5.9 ~ 9.8)	**Emotional control**	8.3, 9.5 (6.7 ~ 9.7)	**Emotional control**	8.3, 8.5 (6.6 ~ 9.9)
10	***Usual activities***	8.2, 9.0 (6.3 ~ 9.9)	**Adaptation to society**	8.0, 8.0 (6.3 ~ 9.7)	Appetite	9.0, 9.5 (7.3 ~ 10.6)	**Adaptation to society**	8.6, 8.0 (7.0 ~ 9.9)
11	**Adaptation to society**	8.3, 8.0 (7.1 ~ 9.5)	Social support (relationships)	8.5, 8.0 (7.1 ~ .9.9)	**Adaptation to society**	9.3, 10.0 (7.9 ~ 10.6)	Tiredness/lack of strength	9.2, 9.5 (8.0 ~ 10.3)
12	Social support (relationships)	8.7, 9.0 (7.3 ~ 10.0)	Tiredness/lack of strength	8.8, 9.5 (7.1 ~ 10.3)	Tiredness/lack of strength	9.9, 11.0 (8.8 ~ 10.9)	Social support (relationships)	9.5, 10.0 (8.2 ~ 10.8)
13	Appetite	9.2, 9.5 (7.7 ~ 10.7)	Appetite	9.6, 11.0 (8.1 ~ 10.9)	Social support (relationships)	10.3, 11.0 (8.9 ~ 11.5)	Appetite	10.3, 11.0 (8.8 ~ 11.7)
14	**Climate adaptation**	12.2, 14.0 (10.7 ~ 13.4)	**Climate adaptation**	11.7, 13.0 (10.3 ~ 13.0)	**Climate adaptation**	12.7, 14.0 (11.7 ~ 13.6)	**Climate adaptation**	11.9, 13.0 (10.2 ~ 13.2)

***Bold italics*** means dimensions from EQ-5D, **bold** means culturally specific dimensions. The ranking order is from 1 (the most important) to 14 (the least important). ** means *P* < 0.01

Group 2 (columns 3 and 4) adopted different instructions: Long version versus short version, both using the same label “moderate problems”. The Spearman correlation coefficient of the mean rank in columns 3 and 4 was 0.961 (*P* < 0.01). Moreover, in different groups for two administrations, the spearman correlation coefficients of questionnaire 3 (moderate problems + long version) for first administration in group 2 and questionnaire 4 (moderate problems + short version) for first and second administration in group 4 were 0.934 (*P* < 0.01) and 0.922 (*P* < 0.01), respectively; the spearman correlation coefficients of questionnaire 4 (moderate problems + short version) for first administration in group 2 and questionnaire 4 (moderate problems + short version) for first and second administration in group 4 were 0.946 (*P* < 0.01) and 0.974 (*P* < 0.01) (See Online Resource 4 Table S4).

In total, these results showed that dimension descriptions influenced rankings more than instructions and confirmed that “moderate problems” stabilized the rank order.

### The test–retest reliability of different dimension descriptions in same groups

Based on the results above, variations in instructions showed little impact on the rankings. Therefore, additional experiments using different dimension descriptions were conducted to assess test–retest reliability.

For group level reliability, we calculated the spearman correlation coefficients of mean ranks of two administrations. In group 3, questionnaire 2 employed “problems + short version”, the spearman correlation coefficient of test–retest (columns 1 and 2, Table 5) was 0.871 (*P* < 0.01). In group 4, questionnaire 4 used “moderate problems + short version”, the spearman correlation coefficient (columns 3 and 4, Table 5) was 0.972 (*P* < 0.01).

**Table 5:** The test–retest results of different dimension descriptions using Short Version instruction in same groups

	Group 3 (n = 20): Test–retest reliability (Problems with [dimension]) (${r}_{s}=$ 0.871**)				Group 4 (n = 21): Test–retest reliability (Moderate problems with [dimension] (${r}_{s}=$ 0.972**)			
	First administration		Second administration		First administration		Second administration	
Number	Questionnaire 2: Problems + Short Version	Mean rank, Median rank, 95% Bootstrap CI	Questionnaire 2: Problems + Short Version	Mean rank, Median rank, 95% Bootstrap CI	Questionnaire 4: Moderate problems + Short Version	Mean rank, Median rank, 95% Bootstrap CI	Questionnaire 4: Moderate problems + Short Version	Mean rank, Median rank, 95% Bootstrap CI
1	***Self-care***	4.4, 4.0 (3.1 ~ 6.0)	***Self-care***	4.8, 3.0 (3.1 ~ 6.6)	***Mobility***	3.7, 3.0 (2.6 ~ 5.0)	***Mobility***	3.6, 2.0 (2.4 ~ 5.0)
2	Sleep	5.8, 6.0 (4.5 ~ 7.1)	***Usual activities***	4.9, 4.0 (3.8 ~ 6.2)	***Pain/discomfort***	4.8, 4.0 (3.2 ~ 6.4)	***Self-care***	4.0, 3.0 (2.8 ~ 5.5)
3	***Mobility***	6.2, 6.5 (4.2 ~ 8.2)	Cognition	5.2, 4.5 (4.2 ~ 6.2)	***Self-care***	5.1, 4.0 (3.6 ~ 6.9)	***Pain/discomfort***	5.3, 5.0 (4.0 ~ 6.6)
4	***Usual activities***	6.3, 5.5 (4.6 ~ 8.0)	***Pain/discomfort***	5.4, 6.0 (4.2 ~ 6.7)	Cognition	6.0, 5.0 (4.4 ~ 7.8)	Cognition	5.5, 5.0 (4.1 ~ 6.8)
5	***Pain/discomfort***	6.4, 5.0 (4.8 ~ 8.2)	Sleep	5.4, 5.0 (4.0 ~ 6.9)	Sleep	6.7, 7.0 (5.5 ~ 7.9)	***Usual activities***	5.6, 4.0 (4.0 ~ 7.5)
6	Cognition	6.9, 7.5 (5.4 ~ 8.3)	***Mobility***	5.6, 4.5 (3.9 ~ 7.5)	***Depression/anxiety***	7.1, 7.0 (5.7 ~ 8.6)	Sleep	7.3, 6.0 (5.8 ~ 8.9)
7	**Emotional control**	6.9, 7.0 (5.4 ~ 8.4)	Vision	7.5, 9.0 (5.3 ~ 10.0)	**Emotional control**	7.5, 8.0 (6.5 ~ 8.6)	***Depression/anxiety***	7.5, 8.0 (6.1 ~ 8.9)
8	***Depression/anxiety***	7.6, 7.5 (6.4 ~ 8.9)	**Emotional control**	8.2, 8.0 (6.7 ~ 9.6)	***Usual activities***	7.5, 7.0 (5.7 ~ 9.2)	**Emotional control**	7.9, 9.0 (6.4 ~ 9.1)
9	Social support (relationships)	7.8, 7.5 (6.2 ~ 9.5)	Social support (relationships)	8.4, 9.0 (6.8 ~ 9.8)	Vision	7.6, 7.0 (5.7 ~ 9.6)	Vision	8.3, 8.0 (6.6 ~ 9.9)
10	Vision	8.1, 10.5 (5.8 ~ 10.3)	Appetite	9.4, 10.0 (7.5 ~ 11.3)	**Adaptation to society**	8.7, 9.0 (7.3 ~ 10.0)	**Adaptation to society**	8.3, 9.0 (7.0 ~ 9.8)
11	**Adaptation to society**	8.8, 9.5 (7.4 ~ 10.2)	***Depression/anxiety***	9.5, 9.5 (8.5 ~ 10.6)	Social support (relationships)	8.9, 9.0 (7.5 ~ 10.2)	Social support (relationships)	8.9, 9.0 (7.6 ~ 10.1)
12	Tiredness/lack of strength	9.2, 9.5 (7.6 ~ 10.6)	**Adaptation to society**	9.9, 10.0 (8.4 ~ 11.1)	Tiredness/lack of strength	9.4, 10.0 (7.8 ~ 10.8)	Tiredness/lack of strength	9.4, 10.0 (8.1 ~ 10.6)
13	Appetite	10.0, 10.0 (8.4 ~ 11.3)	Tiredness/lack of strength	9.9, 10.0 (8.4 ~ 11.2)	Appetite	10.6, 11.0 (9.1 ~ 11.8)	**Climate adaptation**	11.5, 14.0 (9.8 ~ 13.1)
14	**Climate adaptation**	11.0, 11.5 (9.5 ~ 12.3)	**Climate adaptation**	11.1, 11.0 (9.9 ~ 12.3)	**Climate adaptation**	11.5, 13.0 (10.0 ~ 12.9)	Appetite	11.9, 12.0 (11.1 ~ 12.6)

***Bold italics*** means dimensions from EQ-5D, **bold** means culturally specific dimensions. The ranking order is from 1 (the most important) to 14 (the least important). ** means *P* < 0.01

**Fig. 1:**
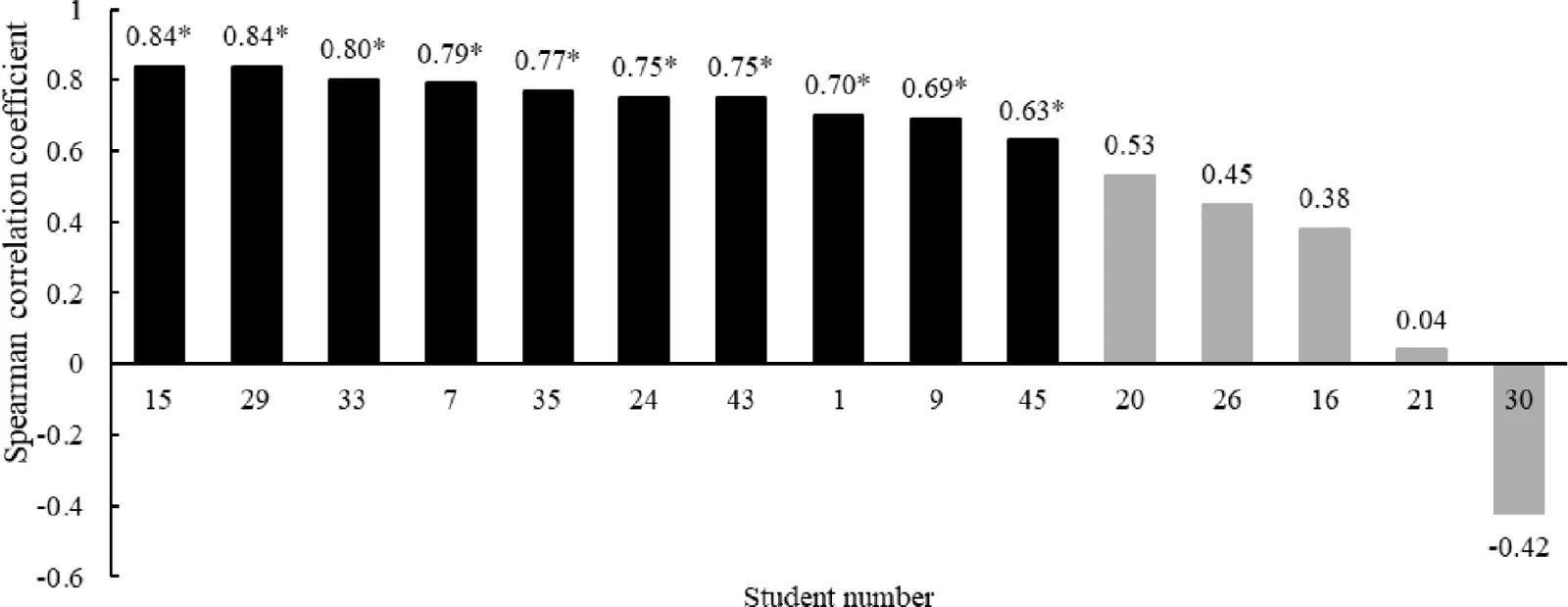
The test–retest individual-level reliability of questionnaire 2. *Note*: * means *P* < 0.05

**Fig. 2:**
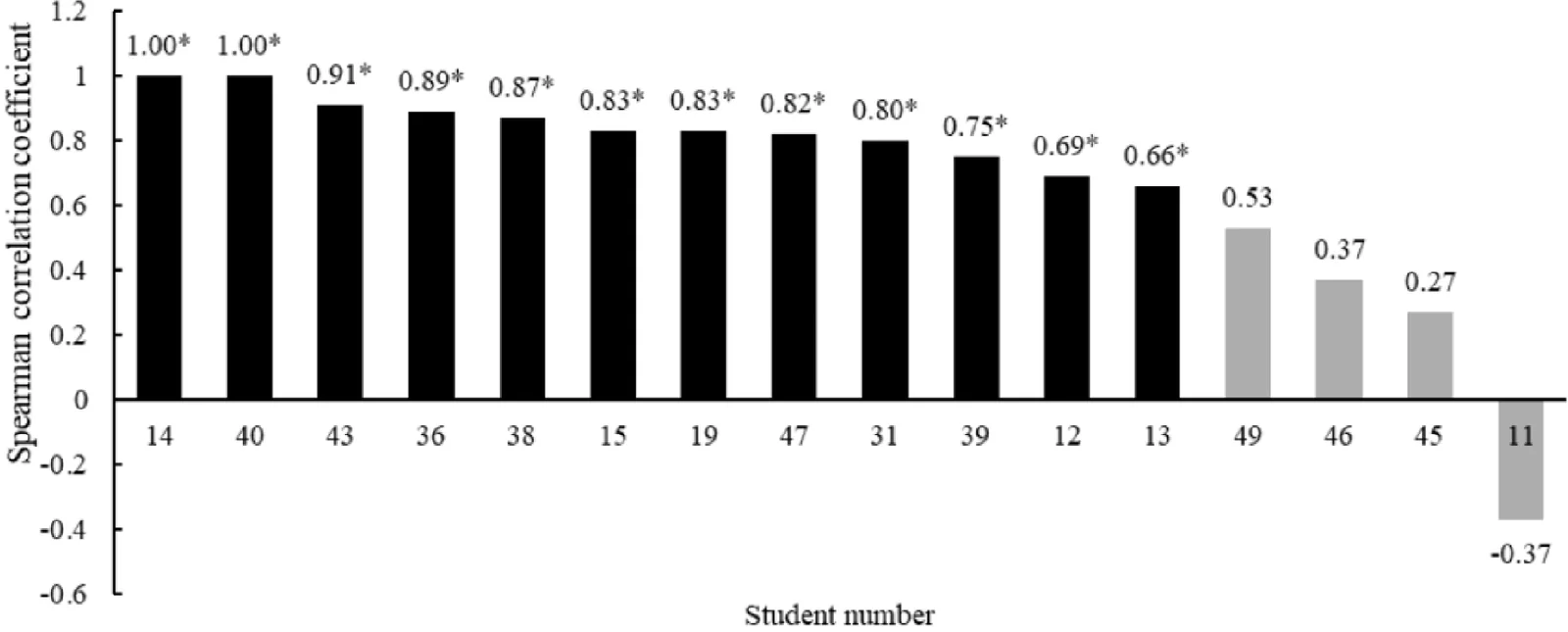
The test–retest individual-level reliability of questionnaire 4. *Note*: * means *P* < 0.05

We also analyzed the individual level reliability for two administrations. Since we only asked the students to enter the last two digits of the student number, and there were overlapping digits across different classes, we were unable to match all responses accurately. Regarding the individual level reliability of questionnaire 2 in group 3, there were 15 matched students. We assessed the spearman correlation coefficient of each individual sorting, the results showed individual level reliability coefficients ranging from − 0.42 to 0.84, See Fig. 1. Additionally, there were 16 matched students in group 4 who completed questionnaire 4, individual reliability coefficients ranged from − 0.37 to 1.00. See Fig. 2. The Media and IQR for questionnaire 2 and questionnaire 4 were 0.70 (0.45 ~ 0.79) and 0.81 (0.56 ~ 0.89), respectively.

These findings suggest that questionnaire 4 (moderate problems + short version) shows higher individual level reliability compared to questionnaire 2 (problems + short version). Thus, “moderate problems” dimension description produced higher test–retest reliability than “problems” description, which means questionnaire 3 and questionnaire 4 produced higher test–retest reliability.

### The ranking order of the dimensions

The rank order of questionnaire 3 in group 2 showed that the five dimensions of EQ-5D, along with sleep and cognition were ranked among the top seven (See Table 3). Additionally, the rank order in questionnaire 4 in group 4 (See Table 3) showed emotional control ranked seventh and usual activity ranked eighth, and the other top six were same with the findings in questionnaire 3.

## Discussion

The present study tested which ranking tasks yielded the most reliable ranks of HRQoL dimensions. Higher consistent results appeared between different instructions, when the moderate dimension descriptions were employed. When both groups used short version instruction, the “moderate problems” description showed higher aggregate stability and preliminary test–retest reliability than “problems” description. These findings suggest that variations in outcomes are primarily influenced by dimension descriptions rather than by the instructions provided.

Ranking is a rudimentary and powerful cognitive process, involving the sorting of items based on perceived importance, preference, or relevance. Similar prioritization processes are performed instinctively by individuals in daily life, like when choosing an outfit, planning daily activities, or selecting a meal from a menu. These routine decisions reflect the natural human tendency to evaluate and order options, demonstrating that ranking is not only a methodological tool in research but also a deeply embedded aspect of human behavior [23].

In our study, variations in instructions had little influence on rankings when both groups adopted the “moderate problems” description. The reason might be that both instruction versions guided participants to rank dimensions by “cure priority”. Indeed, the “moderate problems” description yielded better test–retest reliability and consistency, which was in line with the recommendation that attribute descriptions should be concise and convey concrete meaning [24]. Orme gave the example that it was preferable to specify “weighs 4 kilos” instead of “weighs 3–5 kilos”, because the former was more precise.

Additionally, the wording “problems with [dimension]” is more ambiguous, leaving severity open to interpretation ranging from slight to severe. As the participants reported “problems with vision” can be “slight problems with vision” or “severe problems with vision”. “Slight problems with vision” can be not important, while “severe problems with vision” can be very important. However, the phrase “moderate problems with [dimension]” anchors severity to level 3 of the EQ-5D-5L and helps shift the cognitive frame toward comparable moderate impairments across dimensions, particularly for items such as vision, sleep, and appetite. This severity calibration effect may also account for the better test–retest reliability and consistency using the “moderate problems” description.

In total, the phrasing “moderate problems with [dimension]” was clearer and more specific than “problems with [dimension]”, and the “moderate problems” description appeared more stable for the present methodological purpose. Nevertheless, we should acknowledge that it may be less sensitive in settings where respondents naturally attach greater salience to severe problems.

It may be easy to conclude the added “cultural dimensions” are less relevant to HRQoL than EQ-5D dimensions. However, such conclusion should be approached with caution, as our student sample is not representative of Chinese population.

In this study sample, the EQ-5D dimensions were consistently ranked within the top eight, alongside three additional dimensions: sleep, cognition, and emotional control. Sleep and cognition were classified as “basic physical functions or sensory functions” and “additional culturally universal dimensions”. Physical health was regarded as the foundation that can support other aspects of life and was deemed more important than mental health [25, 26]. As the results showed, although students reported more problems with anxiety/depression, these issues were not ranked highly and remained lower than physical health problems in this sample. The only “additional culturally specific dimension” that ranked ahead of EQ-5D dimensions was “emotional control”, but it still ranked lower than sleep and cognition. A plausible explanation for the relatively high ranking of “emotional control” is its conceptual proximity to anxiety/depression.

In contrast, dimensions such as social adaptation and climate adaptation were ranked lower, possibly due to their perceived limited relevance to health and limited familiarity among participants. These results suggest that “additional culturally specific dimensions” may be not necessarily more relevant than the EQ-5D dimensions—at least not in this sample [6].

The first limitation was the use of a convenience sample and its homogeneity. These undergraduates were younger, healthier, and possessed higher cognitive abilities, which limits the generalizability towards the general population. Additionally, the large variation of ranking scores reflects the preference heterogeneity among individuals, while from an instrument development perspective, we only need to draw inferences about the preferences at aggregate level. In other words, only the most relevant dimensions at aggregate level will be included in the instrument.

Our main aim was to assess the consistency and test–retest reliability of the ranking tasks, not to compare rankings between different groups. Additionally, our sample included undergraduates from the same university, resulting in high homogeneity in terms of age and education level. This homogeneity mitigates some of the potential confounding bias that non-randomization might otherwise introduce, as the groups are naturally comparable on these key demographic variables. Nevertheless, future research refining these methods should ensure that respondents are randomized across experimental administrations.

Our findings indicated that the “moderate problems” showed higher aggregate stability and preliminary individual test–retest reliability. However, we did not examine other severity levels such as slight, severe, or extreme levels, which represented a limitation. It is plausible that preferences for certain dimensions may vary depending on the severity of the problem. For instance, moderate problems with vision may be considered acceptable, whereas severe impairments could be deemed unacceptable. Moreover, previous research reported that the moderate level was better due to heterogeneous religious beliefs concerning death [23]. In such cases, presenting all dimensions at a comparable severity level (e.g., severe pain, severe mobility issues) may help reduce initial framing effects.

Since only the last two digits of student number were recorded for identification, we were only able to match part of subjects when calculating the test–retest reliability at the individual level. The results can only provide a preliminary assessment of individual test–retest reliability. However, combined with the aggregate level test–retest reliability, the “moderate problems with [dimension]” shows higher reliability.

Although the ranking method offers several advantages, including ease of use, reduced scale bias and efficient preference elicitation, several limitations must be considered [27, 28]:

The ranking method identifies the order of preference but does not capture the magnitude of differences between items. For example, it can indicate which dimension is preferred over others, but not by how much. If the research aims to understand the degree of difference between preferences, methods that provide interval-level data might be more appropriate, such as Discrete Choice Experiments etc.

The ranking method is most effective when evaluating a small number of items. Researchers suggest that five items often strike a balance between discrimination ability and respondent burden. Evaluating a larger number of dimensions can be cognitively demanding and may reduce accuracy. For instance, respondents may find it difficult to differentiate between less-preferred alternatives [29]. As a result, further studies should compare the ranking method with other preference eliciting methods, such as Best Worst Scaling (BWS). BWS is considered to reduce cognitive burden, encourage more discriminating choices, and address scale inequivalence caused by differences in response styles [30].

Although we established a randomized arrangement of dimensions, this arrangement remained constant for all respondents in this study. Participants may have been influenced by the fixed order, which may explain part of observed stability. We recommend further investigations should randomize the arrangement of dimensions for each participant to avoid potential starting-order bias [31].

## Conclusion

Even though this study used a small convenience sample, it demonstrates that the ranking method is feasible. Moreover, variations in the dimension descriptions can influence outcomes. Specifically, the results using “moderate problems with [dimension]” show higher aggregate stability and preliminary individual-level test–retest consistency, when compared to “problems with [dimension].” This methodological research contributes to the development of more valid assessments of whether the prioritization of HRQoL dimensions differs across cultures.

## Supplementary Information

Below is the link to the electronic supplementary material.

Supplementary file1 (DOCX 82 KB)

## Data Availability

Data is available on reasonable request.
