# Supplementary material for: Studying different variations of ranking method to explore health related quality of life dimensions prioritization
Source: Qual Life Res. 2026 Aug 1;35(9):254. doi: 10.1007/s11136-026-04350-5 (PMC13428683; doi:10.1007/s11136-026-04350-5)
Supplement: Supplementary file 1 — Supplementary file1 (DOCX 82 KB) [file 11136_2026_4350_MOESM1_ESM.docx]

**Online resource 1**

**More details of pilot study**

A pilot study was conducted with 17 colleagues aged 22–38 to evaluate the feasibility of ranking method, rather than to draw substantive conclusions about the relative importance of specific dimensions. The sample included nine males and eight females. Six participants held doctoral degrees, five held master’s degrees, five were undergraduate students, and one had a college-level education. Regarding occupation, three were clinical doctors, two were PhD students, three worked in companies, six were undergraduates, and three were teachers.

Then we randomized the pilot study’s initial rankings. Each dimension was assigned a random number generated in Excel. These numbers were then sorted in ascending order, generating a randomized sequence of dimensions.

**Online resource 2**


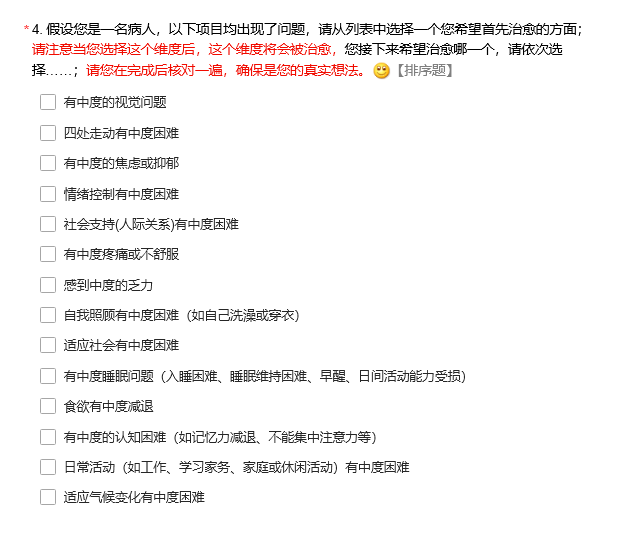


**Figure S1. Screenshot of ranking method on the Wenjuanxing platform**

**Online resource 3**

Questionnaire 1

Instruction

The Long Version instruction: *“Please assume you are a patient and choose one dimension you would prefer to cure first from the list and put that dimension at the top. After you choose the health dimension with problems, assume that the dimension will be cured and assuming the statement will become “have no problems with [that dimension]”. Then choose the next dimension you would prefer to cure, and so on.* *Please review your responses after completion to ensure they reflect your true preferences.”*

1. Problems seeing
2. Problems with walking about
3. Anxiety/depression
4. Problems with emotional control
5. Problems with social support（relationships）
6. Pain/discomfort
7. Tiredness/lack of strength
8. Problems with self-care
9. Problems with adaptation to society
10. Problems with sleep
11. Problems with appetite
12. Problems with cognition
13. Problems with usual activities
14. Problems with climate adaptation

Questionnaire 2

The Short Version instruction: *“Assuming you are a patient, please rank the following health problems in order of priority for cure from* *first to the last.* *Please review your responses after completion to ensure they reflect your true preferences.”*

1. Problems seeing
2. Problems with walking about
3. Anxiety/depression
4. Problems with emotional control
5. Problems with social support（relationships）
6. Pain/discomfort
7. Tiredness/lack of strength
8. Problems with self-care
9. Problems with adaptation to society
10. Problems with sleep
11. Problems with appetite
12. Problems with cognition
13. Problems with usual activities
14. Problems with climate adaptation

Questionnaire 3

The Long Version instruction: *“Please assume you are a patient and choose one dimension you would prefer to cure first from the list and put that dimension at the top. After you choose the health dimension with problems, assume that the dimension will be cured and assuming the statement will become “have no problems with [that dimension]”. Then choose the next dimension you would prefer to cure, and so on.* *Please review your responses after completion to ensure they reflect your true preferences.”*

1. Moderate problems seeing
2. Moderate problems in walking about
3. Moderately anxious/depressed
4. Moderate problems with emotional control
5. Moderate problems with social support（relationships）
6. Moderate pain/discomfort
7. Moderate tiredness/lack of strength
8. Moderate problems with self-care
9. Moderate problems with adaptation to society
10. Moderate problems with sleep
11. Moderate reduction of appetite
12. Moderate problems with cognition
13. Moderate problems with usual activities
14. Moderate problems with climate adaptation

Questionnaire 4

The Short Version instruction: *“Assuming you are a patient, please rank the following health problems in order of priority for cure from first to the last.* *Please review your responses after completion to ensure they reflect your true preferences.”*

1. Moderate problems seeing
2. Moderate problems in walking about
3. Moderately anxious/depressed
4. Moderate problems with emotional control
5. Moderate problems with social support（relationships）
6. Moderate pain/discomfort
7. Moderate tiredness/lack of strength
8. Moderate problems with self-care
9. Moderate problems with adaptation to society
10. Moderate problems with sleep
11. Moderate reduction of appetite
12. Moderate problems with cognition
13. Moderate problems with usual activities
14. Moderate problems with climate adaptation

**Online resource 4**

**Table S1 The EQ-5D-5L and EQ-VAS results of the sample**

| Group | EQ-5D dimensions and EQ-VAS | No problems, n (%) | Slight problems, n (%) | Moderate problems, n (%) | Severe problems, n (%) | Extreme severe problems,  n (%) |
| --- | --- | --- | --- | --- | --- | --- |
| Group 1 | Mobility | 16(80) | 4(20) | 0(0) | 0(0) | 0(0) |
|  | Self-care | 20(100) | 0(0) | 0(0) | 0(0) | 0(0) |
|  | Usual activity | 18(90) | 1(5) | 1(5) | 0(0) | 0(0) |
|  | Pain/discomfort | 14(70) | 6(30) | 0(0) | 0(0) | 0(0) |
|  | Anxiety/depression | 9(45) | 10(50) | 1(5) | 0(0) | 0(0) |
|  | VAS score | 84.05$\pm$9.78 |  |  |  |  |
| Group 2 | Mobility | 18(90) | 2(10) | 0(0) | 0(0) | 0(0) |
|  | Self-care | 20(100) | 0(0) | 0(0) | 0(0) | 0(0) |
|  | Usual activity | 19(95) | 1(5) | 0(0) | 0(0) | 0(0) |
|  | Pain/discomfort | 11(55) | 9(45) | 0(0) | 0(0) | 0(0) |
|  | Anxiety/depression | 8(40) | 12(60) | 0(0) | 0(0) | 0(0) |
|  | VAS score | 82.65$\pm$10.66 |  |  |  |  |
| Group 3 | Mobility | 20(100) | 0(0) | 0(0) | 0(0) | 0(0) |
|  | Self-care | 20(100) | 0(0) | 0(0) | 0(0) | 0(0) |
|  | Usual activity | 20(100) | 0(0) | 0(0) | 0(0) | 0(0) |
|  | Pain/discomfort | 18(90) | 2(10) | 0(0) | 0(0) | 0(0) |
|  | Anxiety/depression | 14(70) | 5(25) | 1(5) | 0(0) | 0(0) |
|  | VAS score | 75.35$\pm$12.26 |  |  |  |  |
| Group 4 | Mobility | 20(95) | 1(5) | 0(0) | 0(0) | 0(0) |
|  | Self-care | 21(100) | 0(0) | 0(0) | 0(0) | 0(0) |
|  | Usual activity | 20(95) | 1(5) | 0(0) | 0(0) | 0(0) |
|  | Pain/discomfort | 13(62) | 8(38) | 0(0) | 0(0) | 0(0) |
|  | Anxiety/depression | 7(33) | 11(53) | 3(14) | 0(0) | 0(0) |
|  | VAS score | 79.71$\pm$19.872 |  |  |  |  |

**Table S2 Proportion of respondents ranking each dimension in the top 5[n(%)]**

| **Number** | **Group 1 (n=20): difference in level description: problems vs moderate problems with [dimension]. Both use the Long Version** | | | | |  | **Group 2 (n=20): difference in instruction: Long Version vs Short Version. Both use moderate problems with [dimension]** | | | | |
| --- | --- | --- | --- | --- | --- | --- | --- | --- | --- | --- | --- |
|  | **First administration** | |  | **Second administration** | |  | **First administration** | |  | **Second administration** | |
|  | **Questionnaire 1:**  **Problems +**  **Long Version** | **Proportion** |  | **Questionnaire 3:**  **Moderate problems +**  **Long Version** | **Proportion** |  | **Questionnaire 3:**  **Moderate problems +**  **Long Version** | **Proportion** |  | **Questionnaire 4:**  **Moderate problems +**  **Short Version** | **Proportion** |
| 1 | Vision | 16(80.0) |  | ***Mobility*** | 14(70.0) |  | ***Mobility*** | 11(55.0) |  | ***Mobility*** | 13(65.0) |
| 2 | ***Mobility*** | 13(65.0) |  | Vision | 12(60.0) |  | ***Pain/discomfort*** | 15(75.0) |  | ***Self-care*** | 13(65.0) |
| 3 | **Emotional control** | 10(50.0) |  | ***Depression/anxiety*** | 8(40.0) |  | ***Self-care*** | 11(55.0) |  | ***Pain/discomfort*** | 14(70.0) |
| 4 | Cognition | 9(45.0) |  | ***Self-care*** | 12(60.0) |  | Sleep | 11(55.0) |  | ***Usual activities*** | 11(55.0) |
| 5 | ***Self-care*** | 10(50.0) |  | ***Pain/discomfort*** | 9(45.0) |  | ***Usual activities*** | 9(45.0) |  | Sleep | 13(65.0) |
| 6 | ***Depression/anxiety*** | 6(30.0) |  | Sleep | 8(40.0) |  | Cognition | 10(50.0) |  | Cognition | 7(35.0) |
| 7 | ***Pain/discomfort*** | 8(40.0) |  | **Emotional control** | 5(25.0) |  | ***Depression/anxiety*** | 9(45.0) |  | ***Depression/anxiety*** | 7(35.0) |
| 8 | Sleep | 6(30.0) |  | Cognition | 4(20.0) |  | Vision | 8(40.0) |  | Vision | 5(25.0) |
| 9 | Tiredness/lack of strength | 7(35.0) |  | ***Usual activities*** | 8(40.0) |  | **Emotional control** | 5(25.0) |  | **Emotional control** | 4(20.0) |
| 10 | ***Usual activities*** | 5(25.0) |  | **Adaptation to society** | 6(30.0) |  | Appetite | 4(20.0) |  | **Adaptation to society** | 3(15.0) |
| 11 | **Adaptation to society** | 2(10.0) |  | Social support (relationships) | 5(25.0) |  | **Adaptation to society** | 3(15.0) |  | Tiredness/lack of strength | 1(5.0) |
| 12 | Social support (relationships) | 2(10.0) |  | Tiredness/lack of strength | 6(30.0) |  | Tiredness/lack of strength | 2(10.0) |  | Social support (relationships) | 3(15.0) |
| 13 | Appetite | 4(20.0) |  | Appetite | 2(10.0） |  | Social support (relationships) | 2(10.0) |  | Appetite | 4(20.0) |
| 14 | **Climate adaptation** | 2(10.0) |  | **Climate adaptation** | 1(5.0) |  | **Climate adaptation** | 0(0.0) |  | **Climate adaptation** | 2(10.0) |

Note: ***Bold italics*** means dimensions from EQ-5D, **bold** means culturally specific dimensions. The ranking order is from 1 (the most important) to 14 (the least important).

**Table S3 Proportion of respondents ranking each dimension in the top 5[n(%)]**

|  | **Group 3 (n=20): test-retest reliability (Problems with [dimension])** | | | | |  | **Group 4 (n=21): test-retest reliability (Moderate problems with [dimension]** | | | | |
| --- | --- | --- | --- | --- | --- | --- | --- | --- | --- | --- | --- |
|  | **First administration** | |  | **Second administration** | |  | **First administration** | |  | **Second administration** | |
| **Number** | **Questionnaire 2:**  **Problems +**  **Short Version** | **Proportion** |  | **Questionnaire 2:**  **Problems +**  **Short Version** | **Proportion** |  | **Questionnaire 4:**  **Moderate problems +**  **Short Version** | **Proportion** |  | **Questionnaire 4:**  **Moderate problems +**  **Short Version** | **Proportion** |
| 1 | ***Self-care*** | 15(75.0) |  | ***Self-care*** | 14(70.0) |  | ***Mobility*** | 17(81.0) |  | ***Mobility*** | 17(81.0) |
| 2 | Sleep | 9(45.0) |  | ***Usual activities*** | 13(65.0) |  | ***Pain/discomfort*** | 13(61.9) |  | ***Self-care*** | 16(76.2) |
| 3 | ***Mobility*** | 9(45.0) |  | Cognition | 14(70.0) |  | ***Self-care*** | 12(57.1) |  | ***Pain/discomfort*** | 12(57.1) |
| 4 | ***Usual activities*** | 10(50.0) |  | ***Pain/discomfort*** | 9(45.0) |  | Cognition | 11(52.4) |  | Cognition | 11(52.4) |
| 5 | ***Pain/discomfort*** | 10(50.0) |  | Sleep | 12(60.0) |  | Sleep | 7(33.3) |  | ***Usual activities*** | 14(66.7) |
| 6 | Cognition | 9(45.0) |  | ***Mobility*** | 11(55.0) |  | ***Depression/anxiety*** | 8(38.1) |  | Sleep | 8(38.1) |
| 7 | **Emotional control** | 6(30.0) |  | Vision | 9(45.0) |  | **Emotional control** | 7(33.3) |  | ***Depression/anxiety*** | 6(28.6) |
| 8 | ***Depression/anxiety*** | 5(25.0) |  | **Emotional control** | 4(20.0) |  | ***Usual activities*** | 7(33.3) |  | **Emotional control** | 5(23.8) |
| 9 | Social support (relationships) | 6(30.0) |  | Social support (relationships) | 3(15.0) |  | Vision | 9(42.9) |  | Vision | 6(28.6) |
| 10 | Vision | 8(40.0) |  | Appetite | 5(25.0) |  | **Adaptation to society** | 3(14.3) |  | **Adaptation to society** | 4(19.0) |
| 11 | **Adaptation to society** | 5(25.0) |  | ***Depression/anxiety*** | 1(5.0) |  | Social support (relationships) | 4(19.0) |  | Social support (relationships) | 2(9.5) |
| 12 | Tiredness/lack of strength | 4(20.0) |  | **Adaptation to society** | 2(10.0) |  | Tiredness/lack of strength | 4(19.0) |  | Tiredness/lack of strength | 2(9.5) |
| 13 | Appetite | 3(15.0) |  | Tiredness/lack of strength | 2(10.0) |  | Appetite | 1(4.8) |  | **Climate adaptation** | 2(9.5) |
| 14 | **Climate adaptation** | 1(5.0) |  | **Climate adaptation** | 1(5.0) |  | **Climate adaptation** | 2(9.5) |  | Appetite | 0(0.0) |

Note: ***Bold italics*** means dimensions from EQ-5D, **bold** means culturally specific dimensions. The ranking order is from 1 (the most important) to 14 (the least important).

**Table S4** **The Spearman correlation analysis of rankings between different questionnaires**

|  |  |  | Group 1 |  | Group 2 |  | Group 3 |  | Group 4 |  |
| --- | --- | --- | --- | --- | --- | --- | --- | --- | --- | --- |
|  |  |  | First administration | Second administration | First administration | Second administration | First administration | Second administration | First administration | Second administration |
|  |  |  | Q 1: Problems +Long Version | Q 3: Moderate problems + Long Version | Q 3: Moderate problems + Long Version | Q 4: Moderate problems +Short Version | Q 2: Problems + Short Version | Q 2: Problems + Short Version | Q 4: Moderate problems + Short Version | Q 4: Moderate problems + Short Version |
| Group 1 | First administration | Q 1: Problems + Long Version | - | - | - | - | - | - | - | - |
|  | Second administration | Q 3: Moderate problems + Long Version | 0.859^**^ | - | - | - | - | - | - | - |
| Group 2 | First administration | Q 3: Moderate problems + Long Version | 0.594^*^ | 0.763^**^ | - | - | - | - | - | - |
|  | Second administration | Q 4: Moderate problems + Short Version | 0.635^*^ | 0.789^**^ | 0.961^**^ | - | - | - | - | - |
| Group 3 | First administration | Q 2: Problems + Short Version | 0.525 | 0.666^**^ | 0.873^**^ | 0.916^**^ | - | - | - | - |
|  | Second administration | Q 2: Problems + Short Version | 0.480 | 0.519 | 0.807^**^ | 0.812^**^ | 0.871^**^ | - | - | - |
| Group 4 | First administration | Q 4: Moderate problems + Short Version | 0.691^**^ | 0.807^**^ | 0.934^**^ | 0.946^**^ | 0.884^**^ | 0.749^**^ | - | - |
|  | Second administration | Q 4: Moderate problems + Short Version | 0.653^*^ | 0.763^**^ | 0.922^**^ | 0.974^**^ | 0.912^**^ | 0.810^**^ | 0.972^**^ | - |

Note: Q means questionnaire, * means *P*<0.05, ** means *P*<0.01.
